# Integrated Proteomic and Transcriptomic Analyses Reveal the Roles of *Brucella* Homolog of BAX Inhibitor 1 in Cell Division and Membrane Homeostasis of *Brucella suis* S2

**DOI:** 10.3389/fmicb.2021.632095

**Published:** 2021-01-28

**Authors:** Guangdong Zhang, Fangli Zhong, Lei Chen, Peipei Qin, Junmei Li, Feijie Zhi, Lulu Tian, Dong Zhou, Pengfei Lin, Huatao Chen, Keqiong Tang, Wei Liu, Yaping Jin, Aihua Wang

**Affiliations:** ^1^College of Veterinary Medicine, Northwest A&F University, Yangling, China; ^2^Key Laboratory of Animal Biotechnology, Ministry of Agriculture, Northwest A&F University, Yangling, China

**Keywords:** *Brucella suis* S2, BAX inhibitor 1 (BI-1), cell viability, cell division, membrane homeostasis, stress resistance, proteomics, transcriptomics

## Abstract

BAX inhibitor 1 (BI-1) is an evolutionarily conserved transmembrane protein first identified in a screening process for human proteins that suppress BAX-induced apoptosis in yeast cells. Eukaryotic BI-1 is a cytoprotective protein that suppresses cell death induced by multiple stimuli in eukaryotes. *Brucella*, the causative agent of brucellosis that threatens public health and animal husbandry, contains a conserved gene that encodes BI-1-like protein. To explore the role of the *Brucella* homolog of BI-1, BrBI, in *Brucella suis* S2, we constructed the *brbI* deletion mutant strain and its complemented strain. *brbI* deletion altered the membrane properties of *Brucella suis* S2 and decreased its resistance to acidic pH, H_2_O_2_, polymyxin B, and lincomycin. Additionally, deleting *brbI* led to defective growth, cell division, and viability in *Brucella suis* S2. We then revealed the effect of *brbI* deletion on the physiological characteristics of *Brucella suis* S2 via integrated transcriptomic and proteomic analyses. The integrated analysis showed that *brbI* deletion significantly affected the expression of multiple genes at the mRNA and/or protein levels. Specifically, the affected divisome proteins, FtsB, FtsI, FtsL, and FtsQ, may be the molecular basis of the impaired cell division of the *brbI* mutant strain, and the extensively affected membrane proteins and transporter-associated proteins were consistent with the phenotype of the membrane properties’ alterations of the *brbI* mutant strain. In conclusion, our results revealed that BrBI is a bacterial cytoprotective protein involved in membrane homeostasis, cell division, and stress resistance in *Brucella suis* S2.

## Introduction

BAX inhibitor 1 (BI-1) is an evolutionarily conserved transmembrane protein, first identified in a screening process for human protein candidates that functionally suppress BAX-induced apoptosis in yeast cells (Huckelhoven, 2004; Henke et al., 2011). Eukaryotic BI-1 is a cytoprotective protein located on the endoplasmic reticulum (ER) membrane with 6-7 transmembrane helices (Chae et al., 2004; Bultynck et al., 2012). In animals, BI-1 exerts its cytoprotective role in the intrinsic apoptotic pathway, which consists of the mitochondria-dependent and ER stress-dependent pathways (Huckelhoven, 2004). In mitochondria-dependent apoptosis, BI-1 interacts directly with the anti-apoptosis proteins, Bcl-2 and Bcl-XL, to inhibit BAX/BAK translocation to the mitochondria, thus protecting cells from mitochondrial dysfunction-induced apoptosis (Huckelhoven, 2004; Liu, 2017). In ER-stress-dependent apoptosis, BI-1 interacts directly with IRE1α, an ER-stress sensor, to reduce BAX and IRE1α binding, thus suppressing ER-stress-induced apoptosis (Chae et al., 2004; Lisbona et al., 2009; Shi et al., 2018). BI-1 also modulates Ca^2+^ homeostasis of the ER, relieving the apoptosis triggered by Ca^2+^ overload (Bultynck et al., 2012; Liu, 2017). Although the mechanism of apoptosis in plants differs from that in animals, plant BI-1 shares a highly conserved structure and function with animal BI-1. In yeast and animal cells, plant BI-1 inhibits mammalian BAX-induced cell death. In plant cells, plant BI-1 suppresses mammalian BAX-induced apoptosis and inhibits cell death triggered by biotic and abiotic stresses such as pathogens, acidic pH, oxidative stress, heat shock, and low nutrition (Watanabe and Lam, 2009).

Automated computational analysis using gene prediction method revealed that BI-1-like proteins also extensively exist in prokaryotes (Gamboa-Tuz et al., 2018). However, only two prokaryotic BI-1 homologs have been reported to date; both share amino acid sequence similarity and concordant hydrophobicity profiles with eukaryotic BI-1 (Kihara et al., 1998; Chang et al., 2014). *Escherichia coli* (*E. coli*) YccA is the first identified prokaryotic member of BI-1 family proteins, which is also a transmembrane protein located on the inner membrane. Although the precise physiological role of YccA in *E. coli* is poorly understood, Kihara et al. (1998, 1999) reported that *E. coli* YccA is a proteolytic substrate of FtsH and shares the same recognition site with some other FtsH substrates. FtsH is a membrane-anchored ATP-dependent metalloprotease, conserved in both prokaryotes and eukaryotes (Schumann, 1999; Langklotz et al., 2012). In prokaryotes, its main role is to degrade redundant or abnormal membrane proteins as part of a quality control mechanism to maintain membrane homeostasis (Schumann, 1999; Langklotz et al., 2012; Bittner et al., 2017). van Stelten et al. (2009) revealed that YccA overexpression in *E. coli* can relieve the bacterial cell death triggered by jamming of the SecY translocation system, a translocator complex consisting of SecY, E, and G (Breukink, 2009). Protein translocation mediated by the SecY translocator is a fundamental process for bacteria (Breukink, 2009). Notably, SecY is an FtsH substrate that shares the same recognition site as YccA, suggesting a bacterial cytoprotective function for YccA (Kihara et al., 1998). *Bacillus subtilis* (*B. subtilis*) YetJ is another prokaryotic BI-1 (Chang et al., 2014). Chang et al. analyzed the crystal structure of *B. subtilis* YetJ and characterized it as a Ca^2+^ homeostasis maintainer, implying that YetJ shares molecularly similar functions to those of eukaryotic BI-1 (Chang et al., 2014). To date, relevant studies on prokaryotic BI-1 remain rare, but suggest that prokaryotic BI-1 may be important for maintaining bacterial cell envelope properties as well as bacterial cell viability. As expected, *Brucella* spp. contain a eukaryotic BI-1 homolog. In the *Brucella suis* S2 (*Brucella suis* bv. 1 str. S2 [*B. suis* S2]) genome, *BSS2_RS00410* is annotated as a gene encoding a BI-1-like protein that remains undefined. Because this BI-1-like protein is 100% conserved in all *Brucella* species, we hereafter refer to this *Brucella* homologue of BI-1 as BrBI.

*Brucella* spp. is Gram-negative, facultative intracellular bacteria belonging to the class Alphaproteobacteria, which can invade various host cells, including macrophages and trophoblasts (Byndloss and Tsolis, 2016). Once inside the host cell, *Brucella* initially suffers harsh intracellular environmental conditions such as low nutrition, acidic pH, and oxidative stress (Gomez et al., 2013; Celli, 2019). As a successful pathogen, *Brucella* has evolved numerous strategies, such as the type IV secretory system (T4SS) encoded by the *virB* operon, the quorum-sensing system, two-component systems, and modified lipopolysaccharide (LPS), to disturb the host immune system and adapt to intracellular environments (Boschiroli et al., 2002; Celli et al., 2003; Uzureau et al., 2007; Martinez-Nunez et al., 2010; Ke et al., 2015; Byndloss and Tsolis, 2016; Altamirano-Silva et al., 2018). Owing to these stealthy strategies, a fraction of internalized *Brucella* survives and multiplies in the host cell, eventually leading to long-term chronic infection. Brucellosis, caused by *Brucella* spp., is a severe globally distributed zoonosis characterized by abortion and infertility in animals and debilitating chronic infections in humans, which threatens public health and sustainable development of animal husbandry worldwide (Byndloss and Tsolis, 2016; Celli, 2019).

Given the membrane-associated role and the bacterial cytoprotective potential of known prokaryotic BI-1, we hypothesized that BrBI may have roles in modulating *B. suis* S2 viability and/or membrane properties. Consequently, we investigated the roles of BrBI in *B. suis* S2 stress tolerance, antibiotic resistance, cell viability, and cell division *via* integrated proteomic and transcriptomic analyses.

## Materials and Methods

### Biosafety Statement

All experiments were performed in accordance with the “Regulations on biosafety of Pathogenic Microorganism Laboratory” (2004) No. 424 set by the State Council of the People’s Republic of China and approved by Biosafety Committee of Northwest A&F University.

### Bacterial Strains and Cultures

The bacterial strains used in this study were derived from the *B. suis* vaccine strain S2 (Di et al., 2016). The strains were cultured in tryptic soy broth (TSB) with shaking or on tryptic soy agar (TSA) at 37°C. To determine the concentrations of viable *B. suis* S2 strains, single bacterial colonies from freshly streaked TSA plates were inoculated into 50 ml TSB and grown at 37°C for 2 days with gentle shaking. Subsequently, the cultures were diluted 100-fold with TSB and incubated at 37°C to exponential phase. The cultures were harvested and resuspended in sterile phosphate-buffered saline (PBS), then serially diluted in sterile PBS and plated onto TSA plates to confirm the concentrations of viable bacteria. Antibiotics, when required, were added at the following concentrations: kanamycin, 50 μg/ml; ampicillin, 100 μg/ml. All work involving live *B. suis* S2 strains was performed in a biosafety level 3 (BSL-3) facility.

### Generation of the *brbI* Deletion Mutant Strain and Its Complemented Strain

To obtain the *brbI* deletion mutant strain, a resistance gene replacement procedure was carried out. Briefly, the upstream homologous arm of *brbI* was amplified using the UP-F and UP-R primers, while the downstream homologous arm of *brbI* was amplified using the DW-F and DW-R primers. A 1375-bp kanamycin-resistance cassette (KanR) fragment was amplified from the pEGFP-C1 plasmid using the KanR-F and KanR-R primers. The KanR fragment and the two homologous arms were then inserted into the pUC18 vector to generate the recombinant pUC18-*brbI*-KanR plasmid which was transfected into electrocompetent *B. suis* S2 cells by electroporation (McQuiston et al., 1995). Transformants were screened in the presence of 50 μg/ml kanamycin. The mutant strain was further confirmed via polymerase chain reaction (PCR) using primers BrBI-TF and BrBI-TR and via quantitative real time polymerase chain reaction (qRT-PCR) using primers BrBI-qF and BrBI-qR.

To obtain the complemented strain, an expression plasmid containing a FLAG-tag (pBBARpc) was constructed (Supplementary Material). The intact *brbI* open reading frame (ORF) was amplified using the BrBI-F/BrBI-R primer pair, and the PCR product was cloned into pBBARpc to generate the recombinant pBBARpc-*brbI* plasmid. Electrocompetent cells of the mutant strain were prepared and electroporated according to standard procedures. Subsequently, transformants were screened in the presence of 100 μg/ml ampicillin. The complemented strain was further confirmed via western blot (WB) using an anti-FLAG antibody (abs830005, Absin, Shanghai, China) and via qRT-PCR using the primers, BrBI-qF and BrBI-qR. The sequences of all the primers used in this study are listed in Table 1.

**TABLE 1:** Primers used in this study.

**Primer**	**Sequence (5′-3′)**
UP-F	GCATGCCAGGTGACGGAGAACATT (*Sph* I, underlined)
UP-R	ACTAGTTGTGATTCATTATGGGGATTTC (*Spe* I, underlined)
DW-F	GTCGACTGGGCAACCGTGAATAATC (*Sal* I, underlined)
DW-R	GAATTCAAGCGCAGCGAATGAAGAT (*Eco*R I, underlined)
KanR-F	GTCGACTCAGGTGGCACTTTTCGGGGA (*Sal* I, underlined)
KanR-R	ACTAGTTTGGGCGTCGCTTGGTCGGT (*Spe* I, underlined)
BrBI-F	GTCGACATGGCTGACTTTCGTAAT (*Sal* I, underlined)
BrBI-R	GAATTCTTTCCGCTGCCCTTATCT (*Eco*R I, underlined)
BrBI-TF	CTTTCCAGCAGCTTCTTTTT
BrBI-TR	CAGCGTCGCCTTCATAGTA
BrBI-qF	CCTTATCCTTGCTTCGGTGG
BrBI-qR	TCCTTGATTTCCTGCGTATCG
orf2-qF	AACCATCACCGCCATCTATAC
orf2-qR	TTTGCCATTTCGTCCATTGTC

### Analysis of the Growth Phenotype of *B. suis* Strains

The three *B. suis* S2 strains were inoculated at equal densities (1 × 10^7^ colony-forming units [CFUs]) into 10 ml of TSB and incubated at 37°C with shaking. The cultures were collected at specific times (0, 8, 16, 24, 32, 40, 48, 56, 64, 72, and 80 h), and the optical density (OD) at 600 nm was measured in a 96-well plate using a microplate reader (Bio-Rad, CA, United States). The three *B. suis* S2 strains were streaked on antibiotic-free TSA plates and incubated at 37°C. After 3 days, the colony sizes of the three *B. suis* S2 strains were measured.

### Bacterial Aggregation Assay

The bacterial aggregation assay was performed as previously reported, with some modifications (Liu et al., 2017). Briefly, the three *B. suis* S2 strains were cultured to the exponential phase as described above, then the cultures were left standing on test-tube racks at room temperature for 24 h. To quantify the bacterial aggregation, the OD_600 nm_ of 100 μl of the culture obtained before and after standing was measured in a 96-well microplate using a microplate reader (Bio-Rad). Percent bacterial aggregation was calculated using the following formula:

(1) %bacterialaggregation=([OD-totalOD]u⁢p⁢p⁢e⁢r⁢p⁢h⁢a⁢s⁢e/OD)total×100

### Scanning Electron Microscopy (SEM) Assay

The three *B. suis* S2 strains were cultured to the exponential phase as described above, then inocula of the three strains were fixed in 2.5% glutaraldehyde solution for 3 h at room temperature. After washing three times with PBS, the bacterial samples were dehydrated in an ascending ethanol gradient (30, 50, 70, 80, 90, 95, and 100% for 15 min each). After critical point drying and gold coating, bacterial cells were observed and imaged using a scanning electron microscope (Regulus8100, Hitachi, Tokyo, Japan).

### Analysis of *B. suis* S2 Strains Viability

The three *B. suis* S2 strains were prepared as described above and adjusted to the same OD_600 nm_ with PBS. The concentrations of viable bacteria from the three *B. suis* S2 strains that had the same OD_600 nm_ were measured by plating onto TSA plates as described above. Propidium iodide (PI) staining was also performed. 2 × 10^7^ CFUs of each bacterial suspension was diluted into 100 μl with PBS and pipetted into separate wells of a 96-well, flat-bottom microplate, then incubated with isovolumetric PI stain solution (20 μM) at room temperature in the dark for 15 min. The fluorescence intensity was then measured (excitation wavelength: 535 nm; emission wavelength: 615 nm) using a fluorescence microplate reader (Spark 10M, TECAN, Männedorf, Switzerland).

### Stress Assay

The three *B. suis* S2 strains were cultured and counted as described above. For the sodium dodecyl sulfate (SDS) sensitivity assay, 10 μl of 10-fold diluted samples from the three *B. suis* S2 strains (10^4^–10^6^ CFUs/ml) were added to a TSA plate containing 0.00325% SDS, then the plate was incubated for 3 days at 37°C.

For the antibiotic-resistance assay, bacterial samples were seeded into a 96-well microplate (2 × 10^4^ CFUs/well) and treated with serially diluted antibiotics (polymyxin B: final concentrations of 1200, 600, 300, 150, 75, 37.5, 18.75, 9.325, and 0 μg/ml; lincomycin: final concentrations of 500, 375, 250, 125, 62.5, 31.25, 15.6, 7.8, 3.9, 1.95, and 0 μg/ml) at 37°C for 1h. The bacterial samples were then diluted 10-fold and plated onto TSA plates, then the plates were incubated for 3 days at 37 °C. The survival rate of each strain was calculated by dividing the antibiotic-treated bacterial CFUs by the untreated bacterial CFUs.

For the environmental stress tolerance assay, samples from the three *B. suis* S2 strains were inoculated into TSB containing H_2_O_2_ (1.25 and 2.5 mM) for 1h and HCl (pH 3.5 and 4.5) for 30 min at 37°C. After treatment, a sample of each strain was diluted serially 10-fold and plated onto TSA plates. The plates were then incubated at 37°C for 3 days and the survival rate of each strain was calculated by dividing the treated bacterial CFUs by the untreated bacterial CFUs.

### RNA Isolation and Quantitative Real-Time PCR

The *B. suis* S2 strains were grown in TSB to the exponential phase, then collected via centrifugation at 4°C. Total RNA was then isolated using TRIzol reagent (Invitrogen, Inc., Carlsbad, CA, United States). The resulting RNA samples were reverse-transcribed into cDNA using HiScript III RT SuperMix (Vazyme, Nanjing, China) per the manufacturer’s recommended protocols. qRT-PCR was then performed using the ChamQ SYBR qPCR Master Mix (Vazyme, Nanjing, China) and Bio-Rad CFX96 Real-Time PCR Systems (Bio-Rad, United States). The 2^–ΔΔ*Ct*^ method was used to analyze the relative transcription levels. The results for each target mRNA were normalized to 16S rRNA transcript levels and averaged.

### Proteomic Profiling

#### Protein Preparation

The bacterial cultures (∼2 × 10^7^ CFUs) were centrifuged at 12,000 × *g* for 5 min at 4°C. The bacterial pellets were then resuspended and homogenized in 300 μl of SDT buffer (4% SDS, 100 mM Dithiothreitol [DTT], 150 mM Tris-HCl pH 8.0). After centrifuging at 14,000 × *g* for 40 min, the supernatant was filtered with 0.22-μm filters and quantified with the BCA Protein Assay Kit (P0012, Beyotime).

#### Protein Digestion and Tandem Mass Tag (TMT) Labeling

Proteins (200 μg per sample) were incorporated into 30 μl SDT buffer. The detergent, DTT and other low-molecular-weight components were removed using UA buffer (8 M urea, 150 mM Tris-HCl pH 8.5) by repeated ultrafiltration (Sartorius, 30 kDa). Next, 100 μl iodoacetamide (100 mM in UA buffer) was added to block reduced cysteine residues. The samples were incubated for 30 min in the dark. The filters were washed three times with 100 μl UA buffer, then twice with 100 μl 0.1 M triethylammonium bicarbonate (TEAB) buffer. Finally, the protein suspensions were digested with 4 μg trypsin (Promega) in 40 μl 0.1 M TEAB buffer overnight at 37°C, and the resulting peptides were collected as a filtrate. The peptide content was estimated via ultraviolet light spectral density at 280 nm using an extinctions coefficient of 1.1 of 0.1% (g/l) solution, which was calculated according to the frequencies of tryptophan and tyrosine in vertebrate proteins. Finally, 100 μg of peptide mixture per sample was labeled using TMT reagent according to the manufacturer’s instructions (Thermo Fisher Scientific).

#### Peptide Fractionation With Reversed Phase Chromatography

TMT-labeled peptides were fractionated by reversed phase chromatography using the Agilent 1260 Infinity II HPLC. The peptide mixture was diluted with buffer A (10 mM HCOONH4, 5% ACN, pH 10.0) and loaded onto a XBridge Peptide BEH C18 Column, 130 Å, 5 μm, 4.6 mm × 100 mm column. The peptides were eluted at a flow rate of 1 ml/min with a gradient of 0%–7% buffer B (10 mM HCOONH4, 85% ACN, pH 10.0) for 5 min, 7–40% buffer B during 5–40 min, 40–100% buffer B during 45–50 min, 100% buffer B during 50–65 min. The elution was monitored at 214 nm based on the ultraviolet light trace, and fractions were collected every 1 min during 5–50 min. The collected fractions were combined into 10 fractions and dried down via vacuum centrifugation at 45°C.

#### Nano Liquid Chromatography-Tandem Mass Spectrometry (LC-MS/MS) Analysis

The peptide mixture was loaded onto the C18-reversed phase analytical column (Thermo Fisher Scientific, Acclaim PepMap RSLC 50 μm × 15 cm, nano viper, P/N164943) in buffer A (0.1% formic acid) and separated with a linear gradient of buffer B (80% acetonitrile and 0.1% formic acid) at flow 300 nl/min. The linear gradient was: 6% buffer B for 5 min, 6–28% buffer B for 63 min, 28–38% buffer B for 10 min, 38–100% buffer B for 7 min, hold in 100% buffer B for 5 min.

#### LC-MS/MS Analysis

LC-MS/MS analysis was performed on a Q Exactive plus mass spectrometer (Thermo Fisher Scientific) coupled to Easy nLC (Thermo Fisher Scientific) for 90 min. The mass spectrometer was operated in positive ion mode. MS data were acquired using a data-dependent top 10 method that dynamically chose the most abundant precursor ions from the survey scan (350–1800 m/z) for HCD fragmentation. Survey scans were acquired at a resolution of 70000 at m/z 200 with an AGC target of 3e6 and a maxIT of 50 ms. MS2 scans were acquired at a resolution of 17500 for HCD spectra at m/z 200 with an AGC target of 2e5 and a maxIT of 45 ms. The isolation width was 2 m/z. Only ions with a charge state between 2 and 6 and a minimum intensity of 2e3 were selected for fragmentation. Dynamic exclusion for selected ions was 30 s. Normalized collision energy was 30 eV.

#### Data Analysis

MS/MS raw files were processed using MASCOT engine (Matrix Science, London, United Kingdom; version 2.6) embedded into Proteome Discoverer 2.2, and searched against the NCBI database (Accession: NZ_CP006961.1 and NZ_CP006962.1). The search parameters included trypsin as the enzyme used to generate peptides with a maximum of two missed cleavages permitted. A precursor mass tolerance of 10 ppm was specified and 0.05 Da tolerance for MS2 fragments. Except for TMT labels, carbamidomethyl (C) was set as a fixed modification. Variable modifications were Oxidation (M) and Acetyl (Protein N-term). A peptide and protein false discovery rate of 1% was enforced using a reverse database search strategy. Proteins with fold change (FC) >1.5, *p*-value < 0.05 (by Student’s *t*-test), and the false discovery rate (FDR) < 0.05 were considered differentially expressed.

### Transcriptomic Profiling

#### RNA Extraction

Total RNA was extracted from the tissue using TRIzol^®^ Reagent according the manufacturer’s instructions (Invitrogen) and genomic DNA was removed using DNase I (Takara). RNA quality was determined using the 2100 Bioanalyser (Agilent) and quantified using the ND-2000 (NanoDrop Technologies). A high-quality RNA sample (OD260 nm/280 nm = 1.8∼2.2, OD260 nm/230 nm ≥ 2.0, RIN ≥ 6.5, 28S:18S ≥ 1.0) was used to construct the sequencing library.

#### Library Preparation, and Illumina HiSeq Sequencing

RNA-seq strand-specific libraries were prepared using the TruSeq RNA sample preparation kit from Illumina (San Diego, CA, United States), using 5 μg of total RNA. The rRNA by was removed using a RiboZero rRNA removal kit (Epicenter), and fragmented using fragmentation buffer. cDNA synthesis, end repair, A-base addition and ligation of the Illumina-index ed adaptors were performed according to Illumina’s protocol. Libraries were then size selected for cDNA target fragments of 200–300 bp on 2% Low Range Ultra Agarose followed by PCR amplification using Phusion DNA polymerase (NEB) for 15 PCR cycles. After quantified by TBS380, paired-end libraries were sequenced by Illumina NovaSeq 6000 sequencing (150 bp × 2).

#### Read Quality Control and Mapping

The raw paired end reads were trimmed and quality controlled using Trimmomatic with the parameters, SLIDINGWINDOW: 4:15 MINLEN:75 (version 0.36^1^). The clean reads were then separately aligned to the reference genome with orientation mode using Rockhopper software^2^ to perform a computational analysis of the bacterial RNA-seq data. Once input, Rockhopper uses RNA sequencing reads generated by high-throughput sequencing technology, which enables calculating gene expression levels with default parameters.

#### Differential Expression Analysis and Functional Enrichment

To identify differentially expressed genes (DEGs) between the two samples, the expression level for each transcript was calculated using the fragments per kilobase of read per million mapped reads (RPKM) method. edgeR^3^ was used for differential expression analysis. The DEGs between the two samples were selected using the following criteria: (i) the logarithmic fold-change was >2 and (ii) the FDR was <0.05. To understand the functions of the DEGs, Gene Ontology (GO) functional enrichment analysis was performed using Goatools^4^. DEGs were significantly enriched in GO terms when their Bonferroni-corrected *p*-value was < 0.05.

### Bioinformatic Analysis

The amino acid sequences were aligned using Clustal Omega Webservers^5^ (Madeira et al., 2019). The protein transmembrane helices were predicted using TMHMM Server v. 2.0^6^. The BrBI three-dimensional (3D) structure was predicted using the I-TASSER Webserver^7^ (Roy et al., 2010; Yang and Zhang, 2015).

### Statistical Analysis

All experiments were independently repeated at least three times, and the data are expressed as the mean ± standard deviation (SD) of representative experiments performed in triplicate. All data were analyzed with SPSS (SPSS, Inc., Cary, NC, United States). One-way analysis of variance (ANOVA) with Tukey’s post-test was used to analyze differences between groups. A *p*-value < 0.05 was considered statistically significant.

## Results

### BrBI Is Predicted to Be a Transmembrane Protein

**FIGURE 1:**
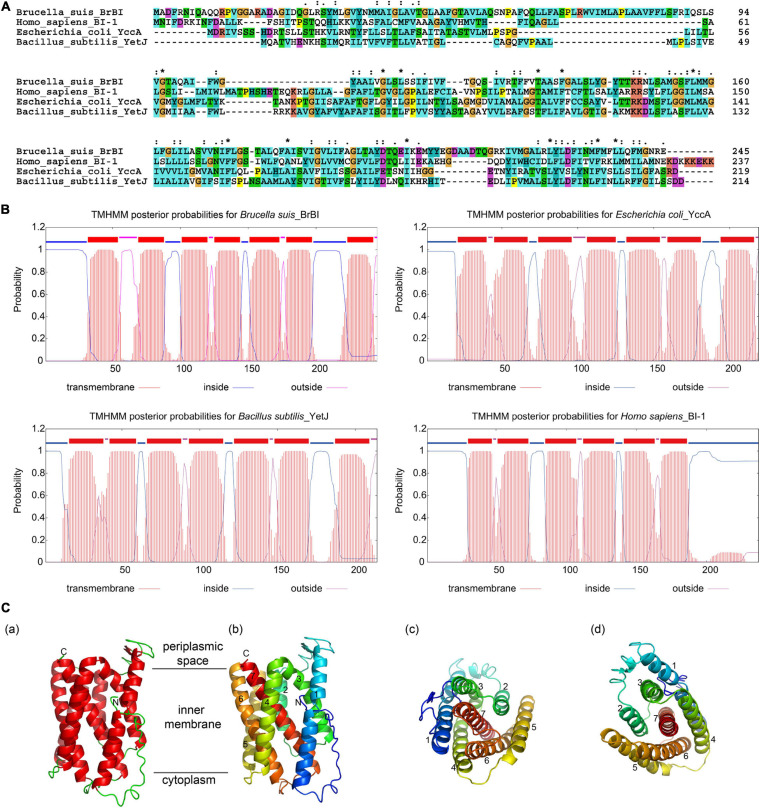
Bioinformatic analysis of BrBI. **(A)** Multiple amino acid sequence alignment of BI-1 family proteins. Orange indicates residue G; yellow indicates residue P; green indicates residues T, S, N, and Q; blue indicates residues W, L, V, I, M, A, F, and C; cyan indicates residues H and Y; magenta indicates residues E and D; red indicates residues K and R; “*” indicates fully conserved residues; “:” indicates strongly conserved residues; “.”indicates weakly conserved residues. **(B)** Prediction of BI-1 family protein transmembrane helices. The protein transmembrane helices were predicted using the TMHMM Server. **(C)** Predictive 3D structure of BrBI by I-TSAAER. **(a)** Red ribbons indicate transmembrane helices; green lines indicate loop structures; N indicates the N-terminus of BrBI; C indicates the C-terminus of BrBI. **(b)** Colored ribbons with numbers indicate 7 transmembrane helices of BrBI. **(c,d)** View of BrBI from the cytoplasm **(c)** and periplasmic space **(d)**. Colored ribbons with numbers indicate 7 transmembrane helices of BrBI. For **(b–d)**, the ribbon was colored using the rainbow color scheme implemented in PyMOL.

As human BI-1 is the first and best characterized eukaryotic BI-1, and *E. coli* YccA and *B. subtilis* YetJ are relatively well-characterized prokaryotic homologs of eukaryotic BI-1, we compared BrBI with these three BI-1 family proteins using bioinformatic methods. BrBI shares 21.09% identity and 47.64% similarity to human BI-1, 21.12% identity and 59.36% similarity to *E. coli* YccA, and 21.43% identity and 47.01% similarity to *B. subtilis* YetJ in amino acid sequences (Figure 1A). Additionally, BrBI contains seven predictive transmembrane helices with a 100% probability, which is concordant with human BI-1, *E. coli* YccA, and *B. subtilis* YetJ (Figure 1B). As human BI-1, *E. coli* YccA, and *B. subtilis* YetJ have been identified as transmembrane proteins, we deduced that BrBI is also a transmembrane protein, and BI-1 family proteins may be structurally conserved. We further predicted the 3D structural model of BrBI. The predicted 3D structure of BrBI contained seven transmembrane segments (Figure 1C) and is homologous to *B. subtilis* YetJ according to the Protein Data Bank library, further revealing that BrBI is a transmembrane protein homologous to other BI-1 family proteins.

### Construction of the *brbI* Mutant Strain and Its Complemented Strain

**FIGURE 2:**
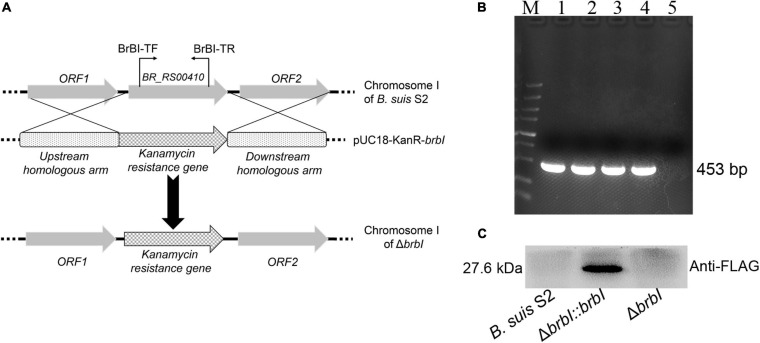
Verification of the Δ*brbI* strain and its complemented strain. **(A)** Schematic of the Δ*brbI* strain generation. **(B)** Δ*brbI* strain verification via PCR using the BrBI-TF/TR primer pair. Lane 1 is the *B. suis* S2 strain; lanes 2–5 are Δ*brbI* strain candidates. Lane 5 is the confirmed mutant strain. **(C)** The Δ*brbI::brbI* strain was verified via WB using a mouse anti-FLAG monoclonal antibody.

To explore the functions of BrBI, we generated a *B. suis* S2 *brbI* deletion mutant strain (Δ*brbI*) via homologous recombination, replacing the intact *brbI* ORF with the kanamycin-resistance gene used as a selection marker (Figure 2A). To further confirm successful generation of the Δ*brbI* strain, we designed a primer pair BrBI-TF/TR residing within the *brbI* ORF (Figure 2A). We randomly selected four kanamycin-resistant transformants for PCR verification and identified a transformant lacking *brbI* (Figure 2B). This *brbI* mutant strain was further confirmed by qRT-PCR (Supplementary Figure S2A). Additionally, qRT-PCR indicated that the deletion of *brbI* did not affect the downstream gene expression (Supplementary Figure S2B). The complemented strain (Δ*brbI::brbI*) was generated via exogenous expression of the intact *brbI* ORF in the Δ*brbI* strain using an expression vector harboring a FLAG-tag. Δ*brbI::brbI* strain generation was confirmed via WB using a mouse anti-FLAG antibody (Figure 2C) and qRT-PCR (Supplementary Figure S2A).

#### Deletion of *brbI* Suppressed *B. suis* S2 Growth

**FIGURE 3:**
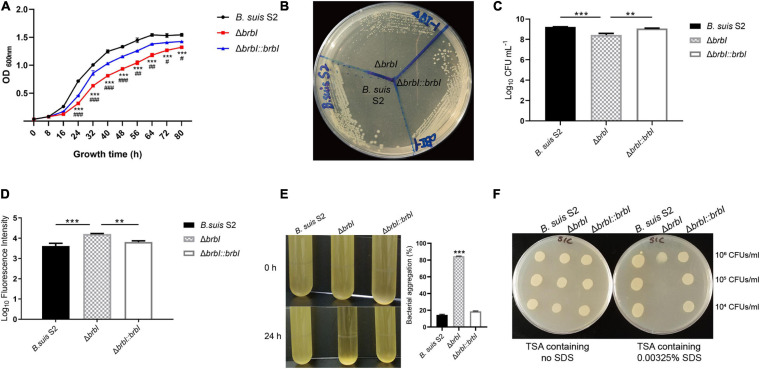
Physiological phenotypes of the Δ*brbI* strain. **(A)** Growth characteristics of the Δ*brbI* strain in TSB. “*” Indicates comparison of the *B. suis* S2 and Δ*brbI* strains; “#” indicates comparison of the Δ*brbI::brbI* and Δ*brbI* strains. **(B)** Growth characteristics of the Δ*brbI* strain on an antibiotic-free TSA plate. **(C)** Viable bacterial concentrations in the three *B. suis* S2 strains with the same OD_600 nm_. The *Brucella* samples were adjusted to the same OD600 nm, then the concentrations of viable bacteria from each sample were measured by plating them onto TSA plates. **(D)** Fluorescence intensity of the three *B. suis* S2 strain samples containing the same number of bacteria. Three *B. suis* S2 strain samples containing 2 × 10^7^ CFUs each were taken and stained with PI, then the fluorescence intensity of each sample was measured. **(E)** Bacterial aggregation phenotype of the Δ*brbI* strain. The three *B. suis* S2 strains were harvested at the exponential phase and left standing at room temperature for 24 h. The OD_600 nm_ of 100 μl of the culture obtained before and after standing was measured to quantify the bacterial aggregation. **(F)** *brbI* deletion increases the sensitivity of *B. suis* S2 to SDS. Ten μl of *Brucella* samples were added to a TSA plate containing 0.00325% or no SDS and incubated for 24 h at 37°C. The data are expressed as the mean ± SD of representative experiments performed in triplicate and analyzed using one-way ANOVA with Tukey’s post-test. ^#^*p* < 0.05,^∗∗^/##*p* < 0.01, ^***^/###*p*< 0.001.

When cultured in TSB, the Δ*brbI* strain displayed a decreased growth rate compared with that of the *B. suis* S2 and Δ*brbI::brbI* strains (Figure 3A). When streaked on an antibiotic-free TSA plate, growth of the Δ*brbI* strain remained significantly impaired, and the Δ*brbI* strain exhibited smaller colonies than did the *B. suis* S2 and Δ*brbI::brbI* strains (Figure 3B). *brbI* deletion clearly suppressed the *B. suis* S2 growth capacity, indicating that deleting *brbI* may affect *B. suis* S2 division and/or survival.

### *brbI* Deletion Impaired *B. suis* S2 Viability

Because *brbI* deletion suppressed *B. suis* S2 growth, and because of the conserved cytoprotective capacity of eukaryotic BI-1 proteins and the bacterial cytoprotective potential of *B. subtilis* YetJ and *E. coli* YccA, we hypothesized that *brbI* deletion might lead to increased cell death in *B. suis* S2. To test this, we investigated how deleting *brbI* would affect the viability of *B. suis* S2. When adjusted to the same OD_600 nm_ (representing the same bacterial cells), the Δ*brbI* strain showed significantly reduced viable bacterial concentrations (represented as CFUs/ml) compared with those of the *B. suis* S2 and Δ*brbI::brbI* strains (reduced ∼4.8- and 3.3-fold, respectively, data not shown), indicating that *brbI* deletion suppressed the survival of *B. suis* S2 (Figure 3C). We performed PI staining to further confirm this. PI is a red fluorescent nucleic acid stain that cannot penetrate intact biological membranes; therefore, the fluorescence intensity of bacteria stained with PI can indicate the cell membrane integrity status. If PI penetrates the membrane, the cell is usually assumed to be dead. When bacterial samples containing 2 × 10^7^ CFUs were stained with PI, the Δ*brbI* strain displayed considerably stronger fluorescence intensity than did either the *B. suis* S2 strain or the complemented strain (increased ∼3.7- and 2.5-fold, respectively, data not shown), indicating a greater proportion of dead bacteria in the Δ*brbI* strain samples (Figure 3D). These results revealed that BrBI is crucial for *B. suis* S2 viability, suggesting that BrBI is a bacterially cytoprotective protein.

### *brbI* Deletion Affected the *B. suis* S2 Membrane Properties

In addition to growth suppression and viability defects, another noticeable phenotype was observed in the Δ*brbI* strain. After standing at room temperature for 24 h, the *B. suis* S2 strain culture harvested at the exponential phase remained uniform, whereas conspicuous bacterial aggregation was observed in the Δ*brbI* strain (Figure 3E). Moreover, *brbI* complementation rescued the bacterial aggregation phenotype. We speculated that *brbI* deletion may have affected the membrane properties of *B. suis* S2. We then performed an SDS-sensitivity assay to determine the membrane properties of the Δ*brbI* strain. As expected, *brbI* deletion significantly increased the sensitivity of *B. suis* S2 to SDS (Figure 3F), suggesting that BrBI may be important for the membrane properties in *B. suis* S2.

### SEM Analysis of the *brbI* Mutant Strain

**FIGURE 4:**
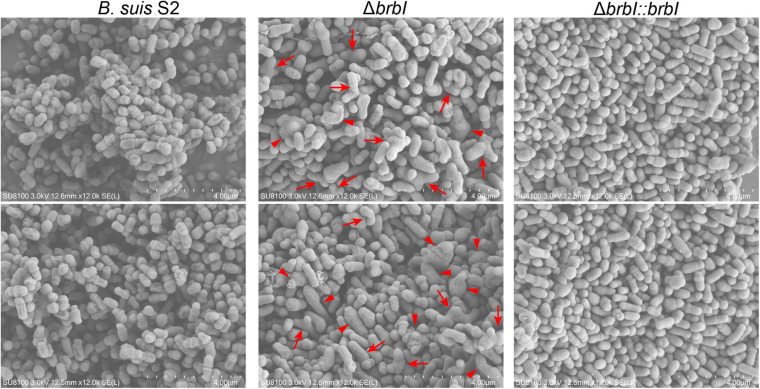
SEM analysis of the Δ*brbI* strain. The *B. suis* S2 strain displayed a typical coccobacillus morphology (short-rod). The morphology of the Δ*brbI* strain was aberrant, and manifested as Y-shaped or branched (arrows), intumesced or elongated bacterial cells (triangles), and fused bacterial cells (star). *brbI* complementation reversed the morphological irregularities. Bar = 4.00 μm.

Scanning electron microscopy analysis was conducted to assess the effect of *brbI* deletion on the *B. suis* S2 morphology (Figure 4). The *B. suis* S2 strain displayed a typical coccobacillus morphology. Conversely, the cell morphology of the Δ*brbI* strain was aberrant: some of the mutant strain showed Y-shaped or branched morphology, while some were intumesced mid-cell, indicating that *brbI* deletion impaired *B. suis* S2 cell division. Complementation of the *brbI* relieved the morphological irregularities. Together, BrBI may involve in the process of cell division in *B. suis* S2, which may contribute to the defective growth and viability in the Δ*brbI* strain.

### *brbI* Deletion Reduced the Stress Resistance of *B. suis* S2

**FIGURE 5:**
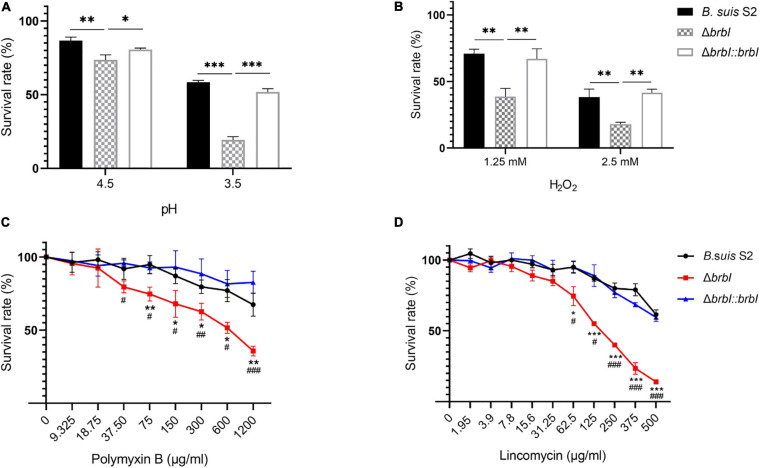
Role of BrBI on stress resistance in *B. suis* S2. Bacterial samples were inoculated into TSB containing HCl **(A)** for 30 min and H_2_O_2_ **(B)** for 1 h at 37°C. For antibiotic resistance, bacterial samples were seeded into a 96-well microplate and treated with serially diluted polymyxin B **(C)** and lincomycin **(D)** at 37°C for 1 h. After treatment, samples were diluted 10-fold and plated onto TSA plates to determine the survival rate of each strain by dividing the treated bacterial CFUs by the untreated bacterial CFUs. In panels C and D, “*” indicates comparison of the *B. suis* S2 and Δ*brbI* strains; “#” indicates comparison of the Δ*brbI::brbI* and Δ*brbI* strains. The data are expressed as the mean ± SD of representative experiments performed in triplicate and analyzed using one-way ANOVA with Tukey’s post-test. ^*/#^*p* < 0.05, ^**/##^*p* < 0.01, ^***/###^*p* < 0.001.

In bacteria, the cell envelope is the first layer to sense environmental stresses. Given the altered membrane properties of the Δ*brbI* strain, we assessed whether *brbI* deletion would affect the sensitivity of *B. suis* S2 to environmental stresses. When exposed to an acidic environment, the survival rate was significantly lower in the Δ*brbI* strain than in the *B. suis* S2 and Δ*brbI::brbI* strains (Figure 5A), indicating that BrBI is crucial for *B. suis* S2 to tolerate acidic stress. When stimulated with H_2_O_2_, cell viability was significantly impaired in the Δ*brbI* strain (Figure 5B), implying that BrBI also promotes *B. suis* S2 tolerance to oxidative stress.

Regarding antibiotic resistance, the bacterial cell envelope is both a barrier to antibiotic penetration and the target of several antibiotics. Hence, we evaluated the impact of *brbI* deletion on the resistance of *B. suis* S2 to polymyxin B and lincomycin. Polymyxin B is a peptide antibiotic that strongly interacts with bacterial membranes and is frequently used to assess the membrane characteristics of *Brucella*. Exposure to polymyxin B concentrations of up to 75 μg/ml significantly decreased the survival rate of the Δ*brbI* strain compared with that of the complemented and *B. suis* S2 strains, indicating increased sensitivity of the Δ*brbI* strain to polymyxin B (Figure 5C). Lincomycin is a lincosamide antibiotic that inhibits bacterial protein synthesis by interacting with the ribosomal 50S subunits. When pre-treated with different lincomycin concentrations for 30 min at 37°C, the *B. suis* S2 and Δ*brbI::brbI* strains displayed similar resistances to lincomycin; however, resistance of the Δ*brbI* strain to lincomycin decreased significantly at 62.5 μg/ml of lincomycin (Figure 5D). Further, *brbI* deletion significantly reduced the MICs of polymyxin B and lincomycin against *B. suis* S2 (Supplementary Figure S3). Thus, BrBI played a crucial role in modulating the sensitivity of *B. suis* S2 to antibiotics.

### Proteomic Analysis

**FIGURE 6:**
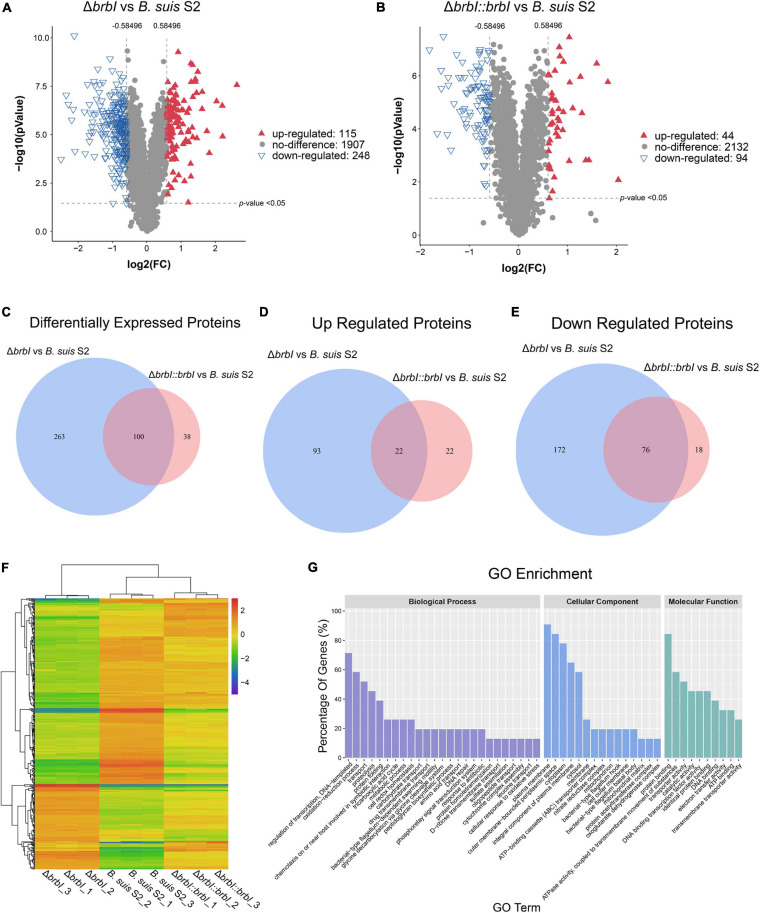
Comparison of the global proteomes of *B. suis* S2, Δ*brbI*, and Δ*brbI::brbI* by LC-MS/MS. **(A)** Volcano plot showing that 363 proteins were significantly differentially expressed in Δ*brbI* compared with *B. suis* S2 with an absolute FC > 1.5 and *p* < 0.05. **(B)** Volcano plot showing that 115 proteins were significantly differentially expressed in Δ*brbI::brbI* compared with *B. suis* S2 with an absolute FC > 1.5 and *p* < 0.05. **(C)** Venn diagram comparing the DEPs between Δ*brbI* and Δ*brbI::brbI*. The blue circle indicates DEPs between *B. suis* S2 and Δ*brbI*; the pink circle indicates DEPs between *B. suis* S2 and Δ*brbI::brbI*; the overlapped area indicates DEPs identified in both Δ*brbI* and Δ*brbI::brbI*. **(D)** Venn diagram comparing the up-regulated proteins between Δ*brbI* and Δ*brbI::brbI*. The blue circle indicates up-regulated proteins between *B. suis* S2 and Δ*brbI*; the pink circle indicates up-regulated proteins between *B. suis* S2 and Δ*brbI::brbI*; the overlapped area indicates up-regulated proteins in both Δ*brbI* and Δ*brbI::brbI*. **(E)** Venn diagram comparing the down-regulated proteins between Δ*brbI* and Δ*brbI::brbI*. The blue circle indicates down-regulated proteins between *B. suis* S2 and Δ*brbI*; the pink circle indicates down-regulated proteins between *B. suis* S2 and Δ*brbI::brbI*; the overlapped area indicates down-regulated proteins in both of Δ*brbI* and Δ*brbI::brbI*. **(F)** Heat map of the two-way hierarchical clustering (436 proteins from three strains satisfying FC > 1.5 using *Z*-score for normalized values [log2 based]). **(G)** GO enrichment analysis of the DEPs between *B. suis* S2 and Δ*brbI* according to the cellular component, molecular function, and biological process categories.

Given the prominent phenotypes induced by *brbI* deletion, we compared the proteomes of the three *B. suis* S2 strains. In total, 2270 proteins were identified in the three *B. suis* S2 strains, and 363 significantly affected proteins were differentially expressed in the Δ*brbI* strain (Figure 6A). Among these differentially expressed proteins (DEPs), 248 were down regulated, and 115 were up regulated. In the complemented strain, 138 proteins were differentially expressed, consisting of 94 down-regulated and 44 up-regulated proteins (Figure 6B). In the Δ*brbI::brbI* strain, complementation of *brbI* recovered 263 DEPs that were identified in the Δ*brbI* strain but induced 38 Δ*brbI::brbI*-specific DEPs, and 100 DEPs were overlapped with the Δ*brbI* strain (Figure 6C). This suggested that *brbI* may have extra functions when expressed beyond physiologic levels. Figures 6C–E show a slight discrepancy in the numbers of overlapped DEPs, attributed to two proteins, BrBI and a DUF1127 domain-containing protein (WP_002965878.1), which were up-regulated in the Δ*brbI::brbI* strain but down-regulated in the Δ*brbI* strain (Supplementary Table S1). Compared with the *B. suis* S2 strain, the remaining 98 overlapped DEPs were regulated in the same directions in the Δ*brbI* and Δ*brbI::brbI* strains. Although these 98 proteins were still differently expressed in the Δ*brbI::brbI* strain compared with the *B. suis* S2 strain, 21 of them were significantly complemented (Supplementary Table S2), showing a 78.24% ([21 + 263]/363) complementation effect. Cluster analysis of 401 DEPs identified in the Δ*brbI* and Δ*brbI::brbI* strains showed a similar result (Figure 6F); these DEPs were divided into Δ*brbI*-specific, Δ*brbI::brbI*-specific, significantly complemented, and uncomplemented DEPs, indicating an acceptable complementation of the complemented strain.

Gene Ontology analysis was performed to further analyse the effects of *brbI* deletion in *B. suis* S2. The 363 DEPs between the Δ*brbI* and *B. suis* S2 strains were enriched to GO terms at the second level in three GO categories: biological process, cellular component and molecular function (Figure 6G and Supplementary Table S3). For biological process, *brbI* deletion mainly affected proteins associated with “regulation of DNA-templated transcription,” “oxidation-reduction,” “transport,” “proteolysis,” and “protein folding.” For cellular component, BrBI was primarily involved in the “plasma membrane,” “outer membrane-bounded periplasmic space,” “cytoplasm,” “integral component of plasma membrane,” and “cytosol.” For molecular function, BrBI mainly regulated proteins associated with “protein binding,” “ATPase activity coupled to transmembrane movement of substances,” “transporter activity,” “catalytic activity,” and “DNA binding transcription factor activity.” In summary, *brbI* deletion predominantly affected proteins associated with the membrane, transporter, and transcription.

### Transcriptomic Analysis

Given the effect of *brbI* deletion on transcriptional activities and because 14 of 363 DEPs identified in the Δ*brbI* strain were transcriptional factors (Supplementary Table S4), we compared the transcriptomes of the three *B. suis* S2 strains.

**FIGURE 7:**
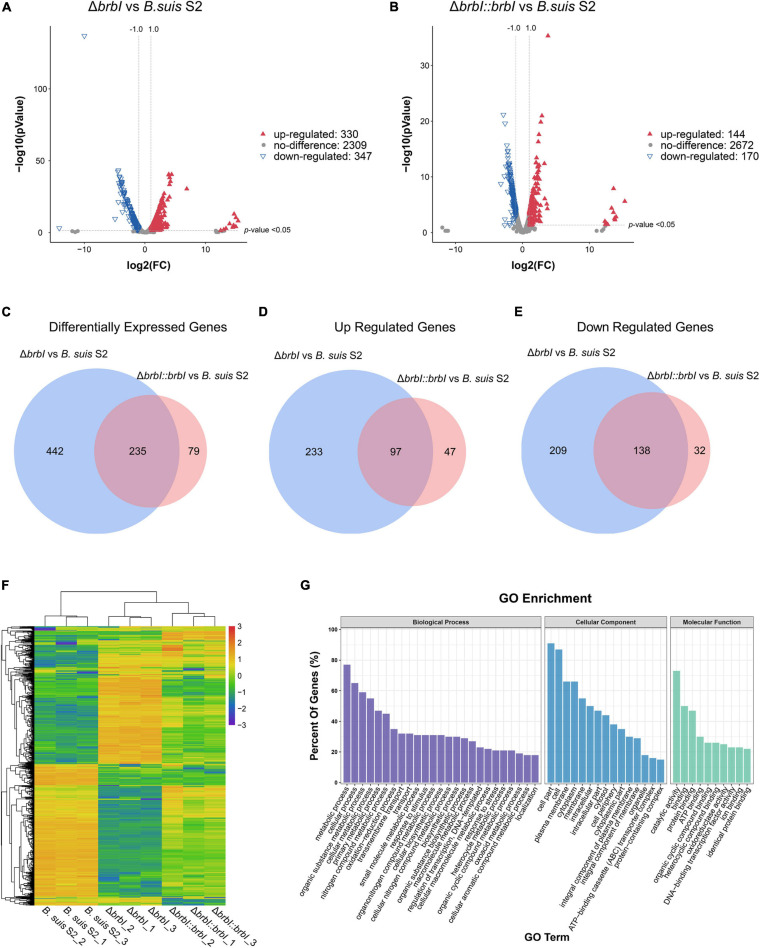
Comparison of the global transcriptomes of *B. suis* S2, Δ*brbI*, and Δ*brbI::brbI* by RNA-sequencing. **(A)** Volcano plot showing that 677 genes were significantly differentially expressed in Δ*brbI* compared with *B. suis* S2 with an FC > 2.0 and *p* < 0.05. **(B)** Volcano plot showing that 314 genes were significantly differentially expressed in Δ*brbI::brbI* compared with *B. suis* S2 with an FC > 2.0 and *p* < 0.05. **(C)** Venn diagram comparing the DEGs between Δ*brbI* and Δ*brbI::brbI*. The blue circle indicates DEGs between *B. suis* S2 and Δ*brbI*; the pink circle indicates DEGs between *B. suis* S2 and Δ*brbI::brbI*; the overlapped area indicates DEGs in both Δ*brbI* and Δ*brbI::brbI*. **(D)** Venn diagram comparing the up-regulated genes between Δ*brbI* and Δ*brbI::brbI*. The blue circle indicates up-regulated genes between *B. suis* S2 and Δ*brbI*; the pink circle indicates up-regulated genes between *B. suis* S2 and Δ*brbI::brbI*; the overlapped area indicates up-regulated genes in both of Δ*brbI* and Δ*brbI::brbI*. **(E)** Venn diagram comparing the down-regulated genes between Δ*brbI* and Δ*brbI::brbI*. The blue circle indicates down-regulated genes between *B. suis* S2 and Δ*brbI*; the pink circle indicates down-regulated genes between *B. suis* S2 and Δ*brbI::brbI*; the overlapped area indicates down-regulated genes in both of Δ*brbI* and Δ*brbI::brbI*. **(F)** Heat map of the two-way hierarchical clustering (754 genes from three strains satisfying an FC > 2.0 using *Z*-score for normalized values [log2 based]). **(G)** GO enrichment analysis of the DEGs between the *B. suis* S2 and Δ*brbI* strains according to the cellular component, molecular function, and biological process categories.

In total, we obtained 14,610,410, 11,764,696, and 12,634,380 clean reads from the *B. suis* S2 strain, 12,396,722, 9,776,270, and 9,215,932 clean reads from the Δ*brbI* strain, and 10,609,342, 18,576,960, and 12,138,906 clean reads from the Δ*brbI::brbI* strain. Further, 87.46–93.46% clean reads were mapped to the *B. suis* S2 reference genome, and 2986 mRNAs were identified in the three *B. suis* S2 strains (Supplementary Table S5). Compared with the *B. suis* S2 strain, 677 genes were significantly differentially transcribed in the Δ*brbI* strain, including 347 down-regulated and 330 up-regulated genes (Figure 7A and Supplementary Table S6). Compared with the *B. suis* S2 strain, 144 up-regulated and 170 down-regulated genes were identified in the Δ*brbI::brbI* strain (Figure 7B and Supplementary Table S6). In the Δ*brbI::brbI* strain, *brbI* complementation recovered 442 DEGs that were identified in the Δ*brbI* strain and induced 79 Δ*brbI::brbI*-specific DEGs, and 235 DEGs were overlapped between the Δ*brbI* and Δ*brbI::brbI* strains (Figures 7C–E). This also suggested that *brbI* may have extra functions when expressed beyond physiologic status. Compared with the *B. suis* S2 strain, the 235 overlapped DEGs were regulated in the same directions in the Δ*brbI* and Δ*brbI::brbI* strains, and 68 of them were significantly complemented in the Δ*brbI::brbI* strain (Supplementary Table S7), displaying a 75.33% ([68+442]/677) complementation effect. Cluster analysis revealed that 756 DEGs identified in the Δ*brbI* and Δ*brbI::brbI* strains could be divided into Δ*brbI*-specific, Δ*brbI::brbI*- specific, significantly complemented, and uncomplemented DEGs (Figure 7F). Considering the stronger sensitivity of transcriptomics to changes, the 75.33% complementation should be an acceptable effect in the complemented strain.

Gene Ontology enrichment analysis showed that the highly enriched GO terms in the Δ*brbI* strain were mainly associated with the biological processes of “metabolic process,” “cellular process,” “organic substance metabolic process,” “cellular metabolic process,” and “primary metabolic process”; the cellular component of “cell part,” “cell,” “plasma membrane,” “cytoplasm,” and “membrane”; the molecular function of “catalytic activity,” “binding,” and “protein binding” (Figure 7G and Supplementary Table S8). These enriched terms were mostly consistent with those in the proteomic profiles with a slight difference in that the predominant genes enriched in the transcriptomic profiles were involved in multiple metabolic processes. This suggested that BrBI played extensive roles in *B. suis* S2 at both the protein and mRNA levels.

### Integrated Analysis Revealed the Global Roles of BrBI in *B. suis* S2

According to the proteomic and transcriptomic profiles, *brbI* deletion extensively affected the physiology of *B. suis* S2 at both the protein and mRNA levels. Therefore, we integrated transcriptomic and proteomic analyses to explore the in-depth effects of *brbI* deletion on *B. suis* S2.

**FIGURE 8:**
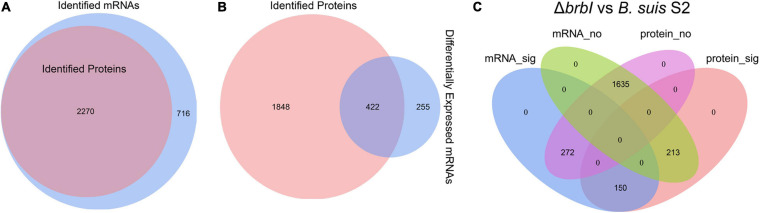
Integrated proteomic and transcriptomic analyses. **(A)** Venn diagram showing that the 2270 proteins identified in the proteomes can all be assigned to the mRNAs identified in the transcriptomes. The blue circle indicates all mRNAs identified in Δ*brbI*; the pink circle indicates all proteins identified in Δ*brbI*. **(B)** Venn diagram showing that 422 of 677 DEGs in the Δ*brbI* strain can be assigned to the 2270 genes identified in both the proteomes and transcriptomes. The blue circle indicates DEGs in Δ*brbI*, the pink circle indicates all proteins in Δ*brbI*; the overlapped area indicates DEGs with corresponding proteins in the proteomic profiles. **(C)** Venn diagram showing the effects of *brbI* deletion on *B. suis* S2 at the mRNA and/or protein levels. The blue ellipse indicates genes significantly affected by *brbI* deletion in the transcriptomic profiles; the pink ellipse indicates genes significantly affected by *brbI* deletion in the proteomic profiles; the green ellipse indicates genes not affected by *brbI* deletion in the transcriptomic profiles; the purple ellipse indicates genes not affected by *brbI* deletion in the proteomic profiles.

As mentioned, 2986 mRNAs and 2270 proteins were identified in the transcriptomic and proteomic profiles, respectively, and these identified proteins could all be assigned to the identified mRNAs (Figure 8A). Thus, we focused on the 2270 genes that were identified in both the transcriptomic and proteomic profiles. Of the DEGs identified in the Δ*brbI* strain, 422 were included in the 2270 genes (Figure 8B). Therefore, 635 genes were significantly affected at the mRNA and/or protein levels in the Δ*brbI* strain (Figure 8C), and we thus targeted these genes going forward. Among the 635 genes, 213 were significantly affected at only the protein level (mRNA_no and protein_sig), 272 were significantly affected at only the mRNA level (mRNA_sig and protein_no), and 150 were significantly affected at both the mRNA and protein levels (mRNA_sig and protein_sig) (Figure 8C and Supplementary Table S4).

The existence of the mRNA_no and protein_sig genes in the Δ*brbI* strain indicated that BrBI participated in the processes of protein translation, stability, and degradation. Because BrBI is a transmembrane protein, and its homolog, *E. coli* YccA, has been characterized as a protease activity-associated protein, we speculated that BrBI may initiate its extensive roles by regulating protein degradation. Consequently, the occurrence of 272 genes being affected at only the mRNA level suggested that some of the DEPs induced by *brbI* deletion should be transcription factors, which was revealed in the proteomic analysis. However, these mRNA_sig and protein_no genes were unaffected by *brbI* deletion at the protein level, indicating that these genes may have a powerful and possibly BrBI-independent protein regulation pathway to overcome the altered gene transcriptions and restore the corresponding proteins to a physiological level. Most of the mRNA_sig and protein_sig genes were regulated in the same directions at the mRNA and protein levels, indicating that these genes should be regulated by *brbI* deletion at only the mRNA level. However, 9 out of 150 mRNA_sig and protein_sig genes showed opposite regulation trends at the mRNA and protein levels. *BSS2_RS10645*, *BSS2_RS10185*, and *BSS2_RS10195* were down-regulated at the mRNA level but up-regulated at protein level, and *BSS2_RS04340*, *BSS2_RS01465*, *BSS2_RS00810*, *BSS2_RS01210*, *BSS2_RS15335*, and *BSS2_RS06495* were upregulated at the mRNA level but downregulated at the protein level (Supplementary Table S4), indicating that they should have a powerful, predominant, and BrBI-dependent protein regulation pathway that can reverse the transcription alterations induced by *brbI* deletion.

According to the known *Brucella* genome annotations, five groups of genes were summarized from the 635 genes discussed here: membrane protein-, cell-division protein-, transcriptional regulator-, peptidase/protease-, and transporter-associated protein-encoding genes (Supplementary Table S4). In total, 27 membrane protein-, 4 cell-division protein-, 31 transcriptional regulator-, 21 peptidase/protease-, and 81 transporter-associated protein-encoding genes were identified. The peptidase/protease- and transporter-associated protein-encoding genes were generally distributed into the mRNA_sig and protein_sig (4 genes and 26 genes, respectively), mRNA_no and protein_sig (14 genes and 25 genes, respectively), and mRNA_sig and protein_no (3 genes and 30 genes, respectively) groups. The transcriptional factor-encoding genes were distributed into the mRNA_sig and protein_no (14 genes) and mRNA_no and protein_sig (17 genes) groups. The membrane protein-encoding genes were mainly distributed into the mRNA_sig and protein_sig (seven genes) and mRNA_no & protein_sig (20 genes) groups. The cell-division protein-encoding genes were specifically distributed into the mRNA_no and protein_sig group.

These results revealed that BrBI plays extensive roles in *B. suis* S2 via protein and/or transcriptional pathways. Importantly, the generally distributed transcriptional factor- and peptidase/protease-encoding genes could provide insights into the underlying mechanisms of BrBI. BrBI may initially modulate transcriptional factors or peptidase/proteases at the protein level, which may further trigger regulations on other regulatory or functional genes at the mRNA and/or protein levels. Notably, the transporter-associated protein-encoding genes were the most abundant and generally distributed, which may explain why *brbI* deletion led to the significantly increased sensitivity of *B. suis* S2 to environmental stresses and antibiotics. Most of the transporter-associated proteins were membrane-related, indicating that BrBI plays a crucial role in the *B. suis* S2 membrane. Further, 27 membrane protein-encoding genes were identified, and interestingly, most were mRNA_no and protein_sig genes. The identified cell-division protein-encoding genes were all mRNA_no and protein_sig genes, indicating a specific role of BrBI in *B. suis* S2 division. Therefore, the role of BrBI in the *B. suis* S2 division and membrane are discussed in detail in the following sections.

### BrBI Is Involved in *B. suis* S2 Division

In Gram-negative bacteria, cell division is tightly mediated by a multi-protein complex called the divisome that is assembled to the mid-cell. FtsZ, FtsA, FtsB, FtsI, FtsL, FtsQ, and FtsW are the primary components of the divisome that execute bacterial cytokinesis (Dajkovic and Lutkenhaus, 2006; Szwedziak and Ghosal, 2017). The divisome is assembled in two stages. First, FtsZ polymers are attached to the mid-cell membrane by interacting with FtsA to form a Z-ring scaffold, which provides the constrictive force. Second, the late-division proteins, including FtsE, FtsK, FtsQ, FtsL and FtsB, FtsW, FtsI, and FtsN, are sequentially recruited to the Z-ring to form the complete septum to carry out cell division (Dajkovic and Lutkenhaus, 2006; Szwedziak and Ghosal, 2017). The early (Z-ring) and late divisome components are linked by the FtsQBL complex, which may have a structural role as a scaffold in assembling the divisome (Choi et al., 2018; Condon et al., 2018). In most bacteria, to guarantee that division occurs at the correct site, a Min system comprising MinC, MinD, and MinE prevents establishing the divisome at bacterial poles (Szwedziak and Ghosal, 2017; Ramm et al., 2019). As a spatial modulator of the divisome, defects in the Min system produce small anucleate minicells from the poles of rod-shaped mother cells, increasing the average cell length of the DNA-containing mother cells (Lutkenhaus, 2007).

**FIGURE 9:**
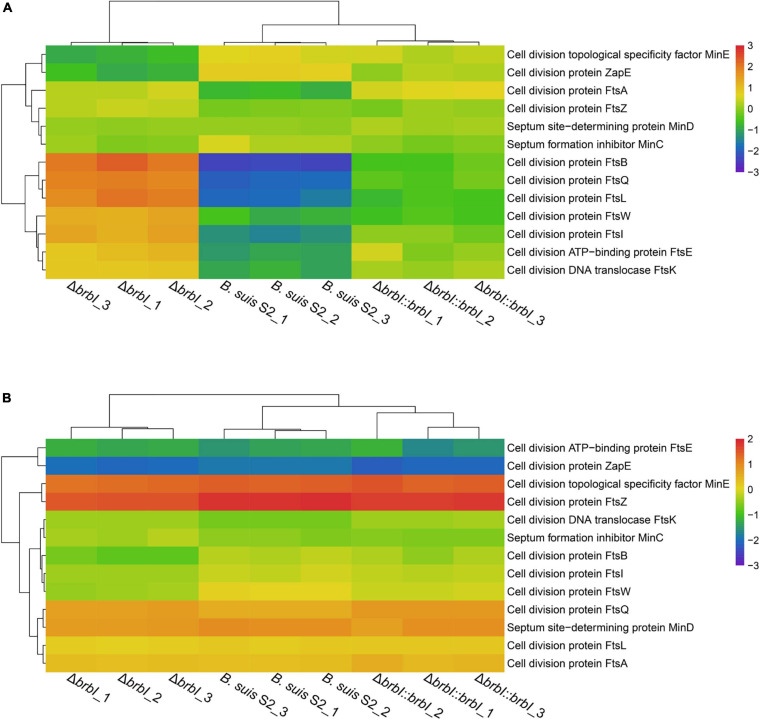
Cluster analysis for cell division-associated genes. **(A)** Cluster analysis for cell division-associated genes at the protein level. **(B)** Cluster analysis for cell division-associated genes at the mRNA level.

As mentioned, *brbI* deletion significantly affected four cell-division protein-encoding genes, indicating a crucial role of BrBI in *B. suis* S2 division. Therefore, we integrated proteomic and transcriptomic analyses to thoroughly explore how *brbI* deletion would affect *B. suis* S2 division. First, *brbI* deletion did not affect FtsZ and FtsA expression at either the mRNA or protein levels, indicating a minor role of BrBI in Z-ring assembly (Figure 9 and Supplementary Table S9). Further, deleting *brbI* did not affect the late divisome proteins, FtsE, FtsK, and FtsW, at either the mRNA or protein levels; however, *brbI* deletion significantly upregulated FtsB, FtsI, FtsL, and FtsQ at only the protein level, possibly indicating disordered cytokinesis septum formation (Figure 9 and Supplementary Table S9). Moreover, *brbI* deletion did not affect MinC, MinD, and MinE expression at either the mRNA or protein levels, indicating a minor role of BrBI in the Min system (Figure 9 and Supplementary Table S9).

In summary, *brbI* deletion may affect proper formation of the division septum, which may further lead to aberrant *B. suis* S2 division. Specifically, the role of BrBI in *B. suis* S2 division may occur predominantly by regulating the translation/degradation/stability of FtsB, FtsI, FtsL, and FtsQ.

### BrBI Is Crucial for *B. suis* S2 Membrane Homeostasis

**FIGURE 10:**
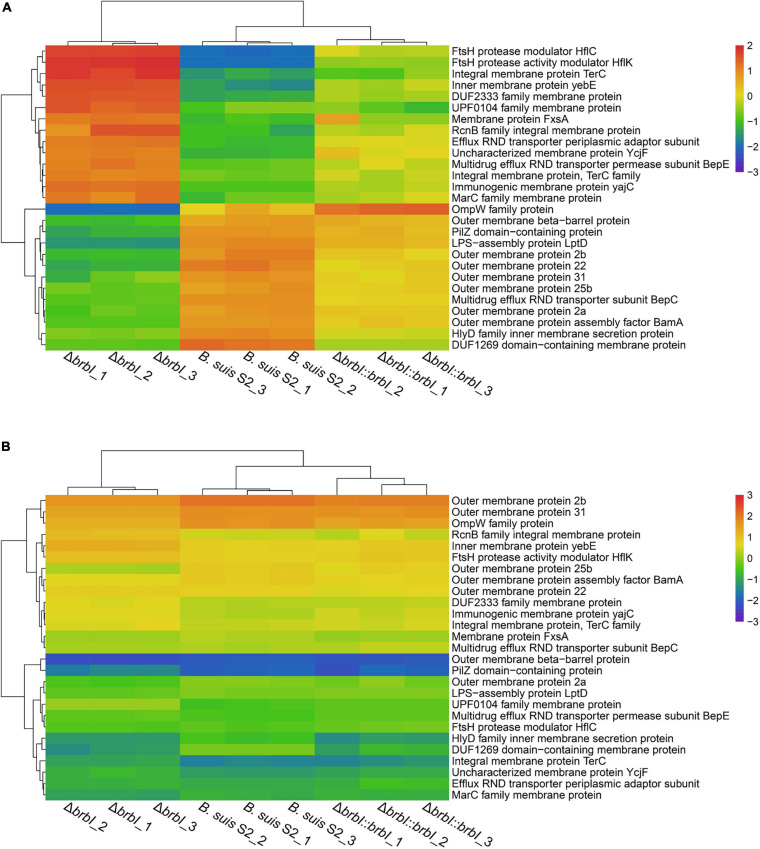
Cluster analysis for membrane protein encoding genes. **(A)** Cluster analysis for membrane protein encoding genes at the protein level. **(B)** Cluster analysis for membrane protein encoding genes at the mRNA level.

The integrated proteomic and transcriptomic analyses showed that *brbI* deletion significantly affected the 27 identified membrane protein-encoding genes (excluding *ftsB*, *ftsI*, *ftsL*, and *ftsQ*); of these, seven were affected at both the mRNA and protein levels, and 20 were affected only at the protein level (Supplementary Table S4). Outer membrane proteins (OMPs) BamA and LptD were significantly down-regulated at only the protein level in the Δ*brbI* strain (Figure 10 and Supplementary Table S9). In Gram-negative bacteria, the lipopolysaccharide transport (Lpt), β-barrel assembly machine (Bam), and the localization of lipoproteins (Lol) pathways are required to transport the outer membrane components (Sperandeo et al., 2019). Lpt, Bam, and Lol are necessary for respectively transporting LPS, β-barrel proteins, and lipoproteins across the periplasm to the outer membrane. Thus, down-regulation of BamA and LptD induced by *brbI* deletion indicated an alteration of the *B. suis* S2 outer membrane. Consistently, the OMP2a, OMP2b, OMP22, OMP25b, OMP31, and OMPW family protein (WP_002964666.1) and outer membrane β-barrel protein (WP_004689965.1) were downregulated at only the protein level with the exception of OMP25b, which was downregulated at both the mRNA and protein levels in the Δ*brbI* strain (Figure 10 and Supplementary Table S9). In *Brucella*, OMP2a and OMP2b are group 2 OMPs, and OMP22, OMP25b, and OMP31 are group 3 OMPs, which are β-barrel proteins (Goolab et al., 2015). Therefore, down-regulation of OMP25b induced by *brbI* deletion may occur at the mRNA level; however, regulation of the remaining OMPs by *brbI* deletion may occur indirectly through BamA at the protein level.

The FtsH protease modulators, HflC and HflK, were also significantly up-regulated at only the protein level via *brbI* deletion (Figure 10 and Supplementary Table S9). FtsH is a membrane-anchored ATP-dependent metalloprotease that degrades redundant or abnormal membrane proteins to maintain membrane homeostasis in prokaryotes (Langklotz et al., 2012; Bittner et al., 2017). HflC and HflK form a membrane protein complex (HflKC) and further complex with FtsH. It has been reported that HflKC may act as a negative modulator for the proteolytic activity of FtsH (Langklotz et al., 2012). Consequently, we speculate that the upregulated HflC and HflK by *brbI* deletion may have suppressed the proteolytic activity of FtsH, subsequently leading to an accumulation of membrane proteins or cytosolic regulatory proteins that were meant to be degraded, which eventually perturbed *B. suis* S2 membrane homeostasis.

Moreover, *brbI* deletion significantly affected several inner membrane proteins and dozens of membrane-related transporter proteins, but the mechanism by which BrBI regulates these proteins remains unknown. Nevertheless, the integrated proteomic and transcriptomic analyses revealed the extensive roles of BrBI in *B. suis* S2 membrane homeostasis. Specifically, OMP was regulated by BrBI predominantly at the protein level.

## Discussion

In this study, we explored the role of BrBI in *B. suis* S2 via basic physiological tests and integrated proteomic and transcriptomic analyses. BrBI played extensive roles in the physiological processes of *B. suis* S2 at both the mRNA and protein levels, including membrane homeostasis, cell division, transport activity, transcription activity, and proteolysis.

Deleting *brbI* in *B. suis* S2 yielded the prominent phenotype of suppressed bacterial growth and viability. Eukaryotic BI-1 has been characterized as a highly conserved cytoprotective protein, although the mechanisms of apoptosis differ between animals and plants (Huckelhoven, 2004; Watanabe and Lam, 2009; Henke et al., 2011; Bultynck et al., 2012). Bacterial (prokaryotic) and eukaryotic BI-1 share a conserved structure (Kihara et al., 1998; Gamboa-Tuz et al., 2018), which is consistent with our finding that BrBI shared highly concordant transmembrane helices with *E. coli* YccA, *B. subtilis* YetJ, and human BI-1. The nature of the cytoprotective role of eukaryotic BI-1 is its function in suppressing cell death, which is not limited to apoptosis and induced by multiple stimuli (Huckelhoven, 2004). It has been hypothesized that BI-1 may be of ancient bacterial origin with a conserved cell viability-associated role (Huckelhoven, 2004; Henke et al., 2011). Our finding that BrBI impairs *B. suis* S2 viability supports this hypothesis. However, the mechanisms of cell death differ between bacteria and eukaryotes, and bacteria do not go through apoptosis. Thus, the mechanism underlying the bacterial cytoprotective role of BrBI remains unclear.

Scanning electron microscopy analysis showed that the Δ*brbI* strain displayed defective cell division, which may have contributed to the *brbI* deletion-induced defects in *B. suis* S2 growth and survival. Our integrated proteomic and transcriptomic analyses revealed that *brbI* deletion did not affect the expressions of FtsA, FtsZ, FtsE, FtsK, FtsW, MinC, MinD, and MinE at either the mRNA or protein levels but specifically increased the protein levels of FtsB, FtsI, FtsL, and FtsQ. Because the FtsQBL complex acts as a scaffold to link the early and late divisome components, these up-regulated proteins may trigger redundant cytokinesis septum formation, subsequently leading to defective cell division (Jain et al., 2018). However, how BrBI regulates division-associated proteins remains unclear. Although defective cell division may be the molecular basis for the suppressed growth and viability of the Δ*brbI* strain, whether BrBI has other mechanisms that modulate *B. suis* S2 viability and/or growth remains unclear.

The integrated proteomic and transcriptomic analyses also showed that *brbI* deletion significantly affected the expression of membrane-associated protein-encoding genes at the mRNA and/or protein levels, such as in the outer membrane protein-, inner membrane protein-, and transporter-associated protein-encoding genes, suggesting a crucial role of BrBI in the *B. suis* S2 membrane properties. The Gram-negative bacterial membrane is multi layered, comprising a symmetric phospholipid bilayer termed the inner membrane, an asymmetric outer membrane containing phospholipids and LPS, and an intermediate aqueous periplasm containing a thin peptidoglycan layer (Hinz et al., 2011; Lehman and Grabowicz, 2019). Regarding integral membrane proteins, inner membrane proteins span the membrane as α-helices, while most OMPs contain a β-barrel structure (Sperandeo et al., 2019). Our results showed that *brbI* deletion significantly affected two key proteins, BamA and LptD, which were down-regulated at only the protein level. In Gram-negative bacteria, Lpt, Bam, and Lol are the three pathways required to respectively transport LPS, β-barrel proteins, and lipoproteins across the periplasm to the outer membrane (Sperandeo et al., 2019). Thus, down-regulation of BamA and LptD by *brbI* deletion suggested a defective assembly of OMPs and LPS in the Δ*brbI* strain. Consistently, seven OMPs were significantly down-regulated. OMP2a, OMP2b, OMP22, and OMP31 were β-barrel containing proteins, which were affected by *brbI* deletion at only the protein level, possibly indicating a link with BamA. Two other key proteins, HflK and HflC, were also significantly affected by *brbI* deletion at only the protein level. Because HflK/C is a negative regulator of FtsH, a critical membrane quality-control mechanism, up-regulation of HflK/C suggested that FtsH may be regulated to alter membrane proteins. However, this hypothesis requires further research. This raises other critical questions. How does BrBI regulate BamA, LptD, HflC, and HflC? Are there other mechanisms by which BrBI regulates membrane proteins? Is there an interaction between BamA and HflK/C? As FtsB, FtsI, FtsL, and FtsQ are also membrane proteins, are they regulated by BamA or HflK/C? Future studies are needed to answer these questions.

*brbI* deletion decreased the resistance of *B. suis* S2 to acid, hydrogen peroxide, polymyxin B, and lincomycin. Although the mechanisms of these stressors in *Brucella* are unclear, the integrated analysis provides insight into how these stressors are affected by *brbI* deletion. First, because the cell envelope is the first layer by which bacteria sense and respond to extracellular stresses, *brbI* deletion-induced membrane disorder may contribute to the increased sensitivity of *B. suis* S2 to stresses. *brbI* deletion significantly downregulated universal stress protein (WP_006191191.1), which may also have led to the decreased resistance of *B. suis* S2 to multiple stresses. Specifically, *brbI* deletion downregulated a series of multidrug efflux RND transporter subunits (WP_011068960.1, WP_004690093.1, and WP_006189806.1), which may participate in reducing antibiotic resistances. Additionally, the acid-activated periplasmic chaperone, HdeA (WP_006191791.1), was significantly decreased in the Δ*brbI* strain, which may contribute to reducing acid resistance.

**FIGURE 11:**
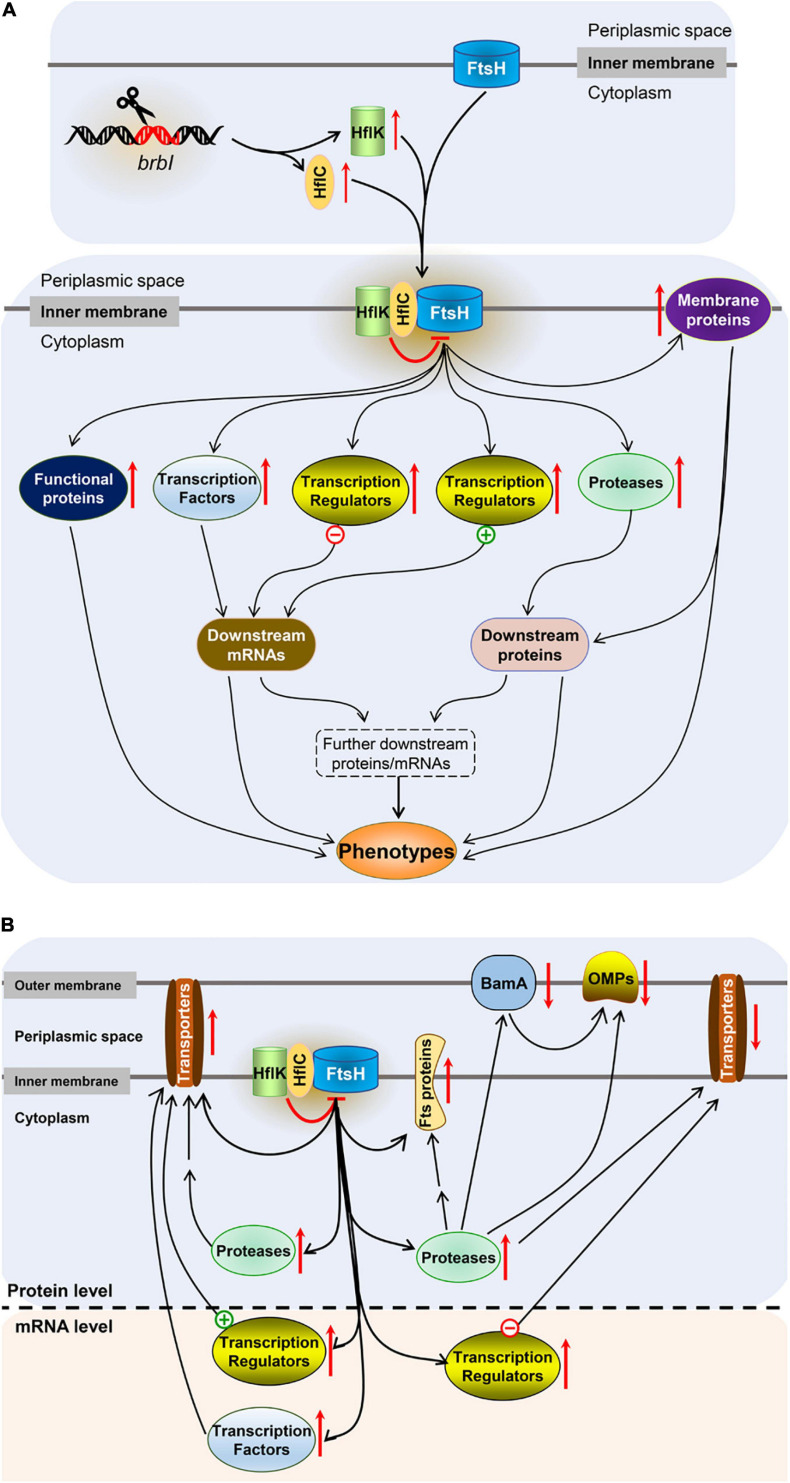
Schematic representation of the major findings. **(A)** The speculated mechanism of BrBI. **(B)** Effects of *brbI* deletion on outer membrane proteins, cell division proteins, and transporter proteins. “–” Indicates negative transcriptional regulators, “+” indicates positive transcriptional regulators, the red arrow indicates the regulation direction of the protein, the double arrow indicates indirect regulation.

As an inner membrane protein, how does BrBI play such an extensive regulatory role? In bacteria, FtsH is the sole ATP-dependent metalloprotease embedded in the plasma membrane (Saikawa et al., 2004) and plays a crucial role in quality control by degrading unneeded or damaged membrane proteins and cytosolic proteins (Bieniossek et al., 2006). Although the degradation mechanism of FtsH remains unclear, a molecular architecture-based model has been proposed. FtsH forms a crown-shaped hexameric molecule consisting of two rings; one is built by the C-terminal protease domains; the other is formed by the AAA (ATPases associated with diverse cellular activities) domains facing the cytosolic leaflet of the membrane (Bieniossek et al., 2006). After the AAA domains bind a recognition tag, ATP hydrolysis leads to translocation of the target polypeptide into the interior of the FtsH “crown,” followed by proteolysis (Bieniossek et al., 2006). HflK and HflC are cytoplasmic membrane proteins and form a complex (Kihara et al., 1996). In *E. coli*, the HflK/C complex directly interacts with FtsH to suppress degradation of several substrates, including SecY, which is a proteolytic substrate of FtsH that shares the same recognition site with YccA (Kihara et al., 1996, 1998). It has been reported that *E. coli* YccA can cross-link with both FtsH and HflK/C, and these four proteins subsequently form an exceptionally large complex in the plasma membrane (Kihara et al., 1998, 1999; Saikawa et al., 2004). YccA11 is a mutant lacking eight amino acids in the N-terminal hydrophilic region of YccA, which can still interact with HflK/C and FtsH but cannot be degraded by FtsH (Kihara et al., 1998, 1999). YccA11 mutation can stabilize excess SecY as does an HflK/C mutation, but deleting the intact YccA cannot accelerate SecY degradation as HflK/C deletion does (Kihara et al., 1998). Moreover, overexpression of HflK/C significantly increased the cross-linking efficiency between YccA11 and FtsH. Kihara et al. concluded that HflK/C was required for YccA to interact with FtsH, and HflK/C acted as a negative regulator for FtsH to degrade specific substrates. Further, the authors speculated that negative regulation of HflK/C for FtsH may attribute to binding of HflK/C and FtsH substrates, which restrained the substrates accessing to the protease domains of FtsH (Kihara et al., 1998; Bieniossek et al., 2006). Combined with the molecular architecture-based model of FtsH proteolysis, the mechanism of HflK/C may be that the HflK/C complex combines with FtsH to increase the spatial proximity of HflK/C and FtsH substrates, then its acting-domain binds with FtsH substrates, restraining the substrates to be translocated into the interior of the FtsH “crown.” As BrBI is homologous to *E. coli* YccA, and FtsH, HflK, and HflC also have chromosomally encoded homologs in *Brucella* spp., we speculate that the possible mechanism of BrBI is that: (i) *brbI* deletion up-regulates HflK and HflC, and the increased HflK/C may suppress FtsH to degrade specific membrane and/or cytosolic proteins, possibly including proteases, transcriptional factors, and other regulatory or functional proteins (such as OMPs, transporter subunits, response regulators, and chaperone proteins); (ii) the accumulated regulatory proteins may further trigger downstream cascade regulation of multiple genes at the mRNA and/or protein levels, ultimately resulting in functional or structural protein alterations; and (iii) alterations of the functional or structural proteins are eventually reflected in their phenotypes (Figure 11A).

In conclusion, mutagenesis and integrated proteomic and transcriptomic analyses revealed that BrBI plays crucial roles in *B. suis* S2 membrane homeostasis, cell viability, and cell division of. Besides, we speculated that as a membrane protein, BrBI may initially regulate specific membrane proteins (such as HflK, HflC, and BamA) at the protein level, then these proteins further regulate multiple gene expressions (may including regulatory, functional, or structural genes) at the mRNA and/or protein levels, and these cascade regulations are eventually reflected on the physiology of *B. suis* S2 (Figure 11B). Nevertheless, because the prokaryotic members of BI-1 family proteins have rarely been studied, the detailed regulatory mechanism of BrBI requires more thorough investigations.

## Data Availability Statement

RNA-seq raw data can be found in the NCBI database, the data was assigned an accession number of GSE160911. The mass spectrometry proteomics data have been deposited to the ProteomeXchange Consortium (http://proteomecentral.proteomexchange.org) via the iProX partner repository with the dataset identifier IPX0002588001/PXD022537.

## Author Contributions

GZ, YJ, and AW conceived and designed the experiments. GZ, FlZ, LC, PQ, JL, FjZ, and LT performed the experiments and analyzed the data. GZ, YJ, and AW wrote and revised the manuscript. DZ, PL, HC, KT, and WL revised the manuscript. All the authors have read and approved the final manuscript.

## Conflict of Interest

The authors declare that the research was conducted in the absence of any commercial or financial relationships that could be construed as a potential conflict of interest.

## Footnotes

1 http://www.usadellab.org/cms/uploads/supplementary/Trimmomatic

2 http://cs.wellesley.edu/∼btjaden/Rockhopper/

3 https://bioconductor.org/packages/release/bioc/html/edgeR.html

4 https://github.com/tanghaibao/Goatools

5 https://www.ebi.ac.uk/Tools/msa/clustalo/

6 https://services.healthtech.dtu.dk/service.php?TMHMM-2.0

7 https://zhanglab.ccmb.med.umich.edu/I-TASSER/
