# Supplementary material for: Integrated Proteomic and Transcriptomic Analyses Reveal the Roles of Brucella Homolog of BAX Inhibitor 1 in Cell Division and Membrane Homeostasis of Brucella suis S2
Source: Front Microbiol. 2021 Jan 28;12:632095. doi: 10.3389/fmicb.2021.632095 (PMC7876416; doi:10.3389/fmicb.2021.632095)
Supplement: Supplementary file 1 [file Data_Sheet_1.zip › Supplementary Material/Table S9_Concerned genes identified in transcriptomic and proteomic profiles.docx]

**Table S8 Concerned genes identified in transcriptomic and proteomic profiles**

| Gene Locus | Protein Accession | Protein Description | Protein | | | | mRNA | | | |
| --- | --- | --- | --- | --- | --- | --- | --- | --- | --- | --- |
|  |  |  | Δ*brbI* vs *B. suis* S2 | | Δ*brbI::brbI* vs *B. suis* S2 | | Δ*brbI* vs *B. suis* S2 | | Δ*brbI::brbI* vs *B. suis* S2 | |
|  |  |  | Fold Change | *p*-value | Fold Change | *p*-value | Fold Change | *p*-value | Fold Change | *p*-value |
| BSS2_RS06450 | WP_004690976.1 | FtsH protease activity modulator HflK | 2.75* | 7.2E-8 | 1.49 | 3.4E-7 | 1.44 | 0.0258 | 1.17 | 0.4386 |
| BSS2_RS06445 | WP_002964505.1 | FtsH protease modulator HflC | 2.69* | 1.9E-8 | 1.63* | 6.1E-5 | 1.35 | 0.0601 | 1.12 | 0.5727 |
| BSS2_RS07950 | WP_004688685.1 | Integral membrane protein TerC | 2.31* | 7.3E-6 | 1.19 | 0.0186 | 1.99 | 0.0001 | 1.20 | 0.3837 |
| BSS2_RS04030 | WP_002964004.1 | Inner membrane protein yebE | 2.27* | 7.5E-7 | 1.39 | 0.0003 | 2.05# | 3.5E-5 | 1.07 | 0.7761 |
| BSS2_RS07270 | WP_002969591.1 | DUF2333 family membrane protein | 2.08* | 5.5E-7 | 1.27 | 0.0002 | 1.54 | 0.0089 | 1.01 | 0.9777 |
| BSS2_RS12535 | WP_002966091.1 | RcnB family integral membrane protein | 1.91* | 0.0013 | 1.27 | 0.0026 | 2.53# | 1.7E-7 | 0.99 | 0.8808 |
| BSS2_RS05055 | WP_006070824.1 | Efflux RND transporter periplasmic adaptor subunit | 1.81* | 1.2E-6 | 1.32 | 1.3E-5 | 1.14 | 0.3259 | 1.39 | 0.0934 |
| BSS2_RS04780 | WP_004690835.1 | Uncharacterized membrane protein YcjF | 1.81* | 8.3E-8 | 1.40 | 0.0007 | 1.69 | 0.0020 | 1.28 | 0.2004 |
| BSS2_RS09525 | WP_002965137.1 | Membrane protein FxsA | 1.78* | 2.6E-6 | 1.29 | 0.1131 | 0.78 | 0.2795 | 0.78 | 0.1630 |
| BSS2_RS04115 | WP_002964021.1 | Immunogenic membrane protein yajC | 1.78* | 2.9E-8 | 1.18 | 0.0001 | 1.71 | 0.0015 | 1.36 | 0.1027 |
| BSS2_RS06145 | WP_006190549.1 | UPF0104 family membrane protein | 1.75* | 6.1E-5 | 0.97 | 0.6542 | 2.48# | 3.2E-7 | 1.29 | 0.1895 |
| BSS2_RS05100 | WP_006190449.1 | MarC family membrane protein | 1.69* | 0.0002 | 1.18 | 0.0165 | 0.75 | 0.2149 | 1.05 | 0.8304 |
| BSS2_RS01370 | WP_006189806.1 | Multidrug efflux RND transporter permease subunit BepE | 1.66* | 1.4E-5 | 1.19 | 0.0027 | 1.40 | 0.0352 | 1.30 | 0.1726 |
| BSS2_RS06770 | WP_004689834.1 | Integral membrane protein, TerC family | 1.60* | 1.9E-6 | 1.14 | 0.0036 | 1.37 | 0.0491 | 1.17 | 0.4207 |
| BSS2_RS04510 | WP_004683739.1 | Outer membrane protein 25b | 0.66* | 0.0002 | 0.84 | 0.0023 | 0.41# | 4.4E-6 | 0.84 | 0.2934 |
| BSS2_RS04905 | WP_006072775.1 | HlyD family inner membrane secretion protein | 0.66* | 2.9E-6 | 0.75 | 1.1E-5 | 0.45# | 5.0E-5 | 0.42# | 2.6E-6 |
| BSS2_RS02945 | WP_006190002.1 | Outer membrane protein 2a | 0.65* | 6.1E-5 | 0.86 | 0.0009 | 0.50 | 0.0006 | 0.90 | 0.4807 |
| BSS2_RS05350 | WP_006190484.1 | Outer membrane protein assembly factor BamA | 0.64* | 1.6E-7 | 0.86 | 0.0002 | 0.68 | 0.0718 | 0.83 | 0.2987 |
| BSS2_RS07475 | WP_004688648.1 | Outer membrane protein 31 | 0.64* | 0.0006 | 0.82 | 0.0004 | 0.60 | 0.0129 | 0.95 | 0.7297 |
| BSS2_RS04360 | WP_011068960.1 | Multidrug efflux RND transporter outer membrane subunit BepC | 0.63* | 4.5E-7 | 0.80 | 5.3E-7 | 0.94 |  | 1.15 | 0.4923 |
| BSS2_RS10380 | WP_004689965.1 | Outer membrane beta-barrel protein | 0.63* | 3.8E-5 | 0.94 | 0.0628 | 0.76 | 0.9317 | 0.88 | 0.4636 |
| BSS2_RS11570 | WP_004688905.1 | DUF1269 domain-containing membrane protein | 0.58* | 4.7E-6 | 0.66* | 8.6E-6 | 0.22# |  | 0.36# | 3.4E-8 |
| BSS2_RS15335 | WP_002966657.1 | PilZ domain-containing protein | 0.58* | 3.8E-6 | 0.90 | 0.0020 | 2.07# | 1E-14 | 0.94 | 0.6935 |
| BSS2_RS02955 | WP_019298809.1 | Outer membrane protein 2b | 0.57* | 6.7E-5 | 0.80 | 0.0023 | 0.52 | 0.0009 | 0.80 | 0.1930 |
| BSS2_RS05940 | WP_004689772.1 | Outer membrane protein 22 | 0.54* | 1.6E-5 | 0.77 | 0.0005 | 1.10 | 0.4322 | 0.84 | 0.3087 |
| BSS2_RS07215 | WP_002964666.1 | OmpW family protein | 0.54* | 0.0005 | 1.31 | 0.0034 | 0.51 | 0.0006 | 0.73 | 0.0792 |
| BSS2_RS03155 | WP_011068938.1 | LPS-assembly protein LptD | 0.51* | 2.5E-6 | 0.88 | 0.0009 | 0.76 | 0.2234 | 1.03 | 0.9228 |
| BSS2_RS05240 | WP_002964259.1 | Cell division protein FtsB | 2.32* | 6.2E-6 | 1.43 | 0.0002 | 0.54 | 0.0028 | 0.88 | 0.4343 |
| BSS2_RS06590 | WP_004691599.1 | Cell division protein FtsQ | 2.03* | 5.5E-7 | 1.33 | 0.0002 | 1.21 | 0.1995 | 1.35 | 0.1100 |
| BSS2_RS06645 | WP_004688556.1 | Cell division protein FtsL | 2.00* | 7.9E-6 | 1.25 | 0.0005 | 0.95 | 0.9942 | 1.04 | 0.8552 |
| BSS2_RS06640 | WP_002964545.1 | Cell division protein FtsI | 1.66* | 8.9E-6 | 1.27 | 0.0004 | 0.74 | 0.1667 | 0.92 | 0.6070 |
| BSS2_RS06615 | WP_002966894.1 | Cell division protein FtsW | 1.47 | 4.3E-5 | 1.04 | 0.1160 | 0.58 | 0.0065 | 0.78 | 0.1557 |
| BSS2_RS09175 | WP_002965062.1 | Cell division ATP-binding protein FtsE | 1.47 | 5.8E-5 | 1.25 | 0.0039 | 1.18 | 0.2503 | 0.98 | 0.8283 |
| BSS2_RS08705 | WP_006190925.1 | Cell division DNA translocase FtsK | 1.40 | 1.1E-5 | 1.22 | 0.0004 | 1.34 | 0.0659 | 1.38 | 0.2740 |
| BSS2_RS06585 | WP_002964534.1 | Cell division protein FtsA | 1.24 | 0.0002 | 1.29 | 6.4E-5 | 1.00 | 0.7821 | 1.21 | 0.3237 |
| BSS2_RS06580 | WP_004690984.1 | Cell division protein FtsZ | 1.10 | 0.0004 | 1.03 | 0.2112 | 0.68 | 0.0644 | 0.88 | 0.4675 |
| BSS2_RS11500 | WP_002966267.1 | Cell division topological specificity factor MinE | 0.76 | 0.0001 | 0.95 | 0.0586 | 0.83 | 0.4522 | 1.05 | 0.8549 |
| BSS2_RS11495 | WP_006073438.1 | Septum site-determining protein MinD | 0.99 | 0.4970 | 1.03 | 0.0106 | 0.86 | 0.5941 | 0.92 | 0.6376 |
| BSS2_RS11490 | WP_004692436.1 | Septum formation inhibitor MinC | 0.94 | 0.0442 | 0.92 | 0.0208 | 1.29 | 0.1028 | 0.95 | 0.7436 |

Note: * represents significantly differentially expressed protein (FC >1.5 or FC <0.67); # represents significantly differentially expressed gene (FC >2.0 or FC <0.50).
