# Supplementary material for: Integrated Proteomic and Transcriptomic Analyses Reveal the Roles of Brucella Homolog of BAX Inhibitor 1 in Cell Division and Membrane Homeostasis of Brucella suis S2
Source: Front Microbiol. 2021 Jan 28;12:632095. doi: 10.3389/fmicb.2021.632095 (PMC7876416; doi:10.3389/fmicb.2021.632095)
Supplement: Supplementary file 1 [file Data_Sheet_1.zip › Supplementary Material/Supplementary Materials.doc]

Supplementary Material

**Construction of pBBARpc**

The pBBARpc plasmid used for complementation of *Brucella* BI-1 was derived from broad-host-plasmid pBBR1MCS-5. pBBR1MCS-5 was used as the template to amplify plasmid replication-associated genes using primers REP-F (ACCGGTCATATGAAAGTCGTGACCGCCTAC, *Nde* I) and REP-R (CTCGAGAAGATGCATTGCTGCGCAACCCAAGTG, *Nsi* I), and pc promoter region harboring FLAG-tag using primers pGMR-F (CCAATGCATAGGCACGAACCCAGTTGACATAA, *Nsi* I) and pGMR-R (GCTCTAGACTTGTCGTCGTCGTCCTTATAGTCCATCGTTGCTGCTCCATAACATCAA, *Xba* I). pET32a was used as the template to amplify ampicillin-resistant gene cassette using primers AmpR-F (catatgTCAGGTGGCACTTTTCGGGGA, *Nde* I) and AmpR-R (CTCGAGACAGTTACCAATGCTTAATCAGTGAGG, *Xho* I), Besides, a multiple cloning sites (MCS) region harboring T7 and M13F primers was designed as MCS-F (CTAGAGTCGACGGATCCGGTACCGAATTCAGGCTTGAGCTCCAATTCGCCCTATAGTGAGTCGTATTACGCGCGCTCACTGGCCGTCGTTTTACC), MCS-R (TCGAGGTAAAACGACGGCCAGTGAGCGCGCGTAATACGACTCACTATAGGGCGAATTGGAGCTCAAGCCTGAATTCGGTACCGGATCCGTCGACT). Subsequently, the three PCR products and the annealed MCS region were ligated together to generate the pBBARpc plasmid.

**Figure S1.** Construction of the pBBARpc plasmid. **(A)** The map of the pBBARpc. **(B)** The verification of the pBBARpc plasmid by WB using anti-FLAG antibody.

**Figure S2.** Verification of the Δ*brbI* strain *via* qRT-PCR. **(A)** No *brbI* mRNA was detected in the Δ*brbI* strain. The *brbI* mRNA level in the Δ*brbI::brbI* strain was significantly increased compared with that of the *B. suis* S2 strain. **(B)** The downstream gene of *brbI* was not affected by the deletion of *brbI*. ***p*<0.01; ns = non-significant

**Figure S3.** Minimal inhibitory concentrations (MIC) of polymyxin B and lincomycin against the Δ*brbI* strain. In a 96-well plate, 10^6^ CFUs of each strain were used to inoculate 200 µl TSB containing two-fold dilutions of polymyxin B (ranged from 0.293 μg/ml to 150 μg/ml) and lincomycin (ranged from 0.195 μg/ml to 100 μg/ml), and the MICs were determined after 24 h of incubation at 37 °C. The black arrow indicates the MIC against the *B. suis* S2 strain; the blue arrow indicates the MIC against the Δ*brbI::brbI* strain; the red arrow indicates the MIC against the Δ*brbI* strain.
